# How can you manage an indomethacin-responsive headache in someone who cannot take indomethacin?

**DOI:** 10.1097/WCO.0000000000001347

**Published:** 2025-02-05

**Authors:** Aleksander Osiowski, Kacper Stolarz, Dominik Taterra

**Affiliations:** a Faculty of Medicine, Jagiellonian University Medical College, Cracow; b Department of Orthopedics, Jagiellonian University Medical College, Zakopane, Poland

**Keywords:** hemicrania continua, indomethacin-responsive headache, paroxysmal hemicrania, side effects, treatment recommendations

## Abstract

**Purpose of review:**

Paroxysmal hemicrania and hemicrania continua are rare primary headache disorders which are distinguished by an absolute response to indomethacin. As a matter of importance, no guidelines have been proposed for alternative therapeutic options in case of indomethacin intolerance. The purpose of this review is to provide an update on the current findings, especially focusing on the past 18 months, in the treatment of both paroxysmal hemicrania and hemicrania continua and to provide proposed management recommendations based on summarized evidence.

**Recent findings:**

Apart from well recognized gastrolesive effects of indomethacin, a substantial number of patients may suffer from neuropsychiatric adverse reactions. Recent studies demonstrated that melatonin, which has been known for its effectiveness for hemicrania continua, is also useful for paroxysmal hemicrania. Promising nonpharmacological treatment option, which is noninvasive vagus nerve stimulation, has been shown to be beneficial for both indomethacin-responsive headache disorders allowing the reduction of indomethacin dosage. Although the data on substitutive medication choice for indomethacin are currently scarce, the most consistent results have been repeatedly achieved with acemethacin, selective COX-2 inhibitors, and anticonvulsants. However, considering the crucial role of pathophysiology, research investigating the efficacy of drugs targeting the trigemino-vascular system activation, as well as controlled trials assessing the efficacy involving the aforementioned therapeutic options are still vague.

**Summary:**

In spite of numerous reports suggesting reliable alternatives to indomethacin, the consensus on pharmacological therapy guidelines for indomethacin-responsive headache disorders has not yet been reached. Further research and agreement from the experts’ standpoint are needed for an establishment of reliable treatment recommendations.

## INTRODUCTION

Indomethacin-responsive headache disorders is a group of unique entities which are distinguished by robust and often absolute response to therapeutic doses of indomethacin. Certain cephalalgias such as primary stabbing headache, hypnic headache, and valsalva-induced headaches tend to differ substantially in terms of the efficacy of indomethacin. Interestingly, according to the International Classification of Headache Disorders, 3rd edition [1] (ICHD-III), two distinct trigeminal-autonomic cephalalgias (TACs), paroxysmal hemicrania and hemicrania continua, are defined by the compulsory response to this medicine. It can be confidently acknowledged that both disorders are relatively rare encounters, as recent meta-analyses estimated that the relative frequency of PH and HC among adult patients evaluated for a headache in tertiary-care center settings was 0.3 and 1.8%, respectively [2^▪^,3^▪^]. A recent Norwegian population-based study estimated 1-year prevalence for hemicranias, which was 1.4 for paroxysmal hemicrania and 2.2 for hemicrania continua per 100 000 individuals [4]. Therefore, patients in whom indomethacin trial results in complete control of their headache attacks definitely praise the knowledgeable clinician for their prominent response [5]. As a matter of fact, this exceptional feature of indomethacin-responsive TACs is indeed what impressively defines them [6]. 

**Box 1:**
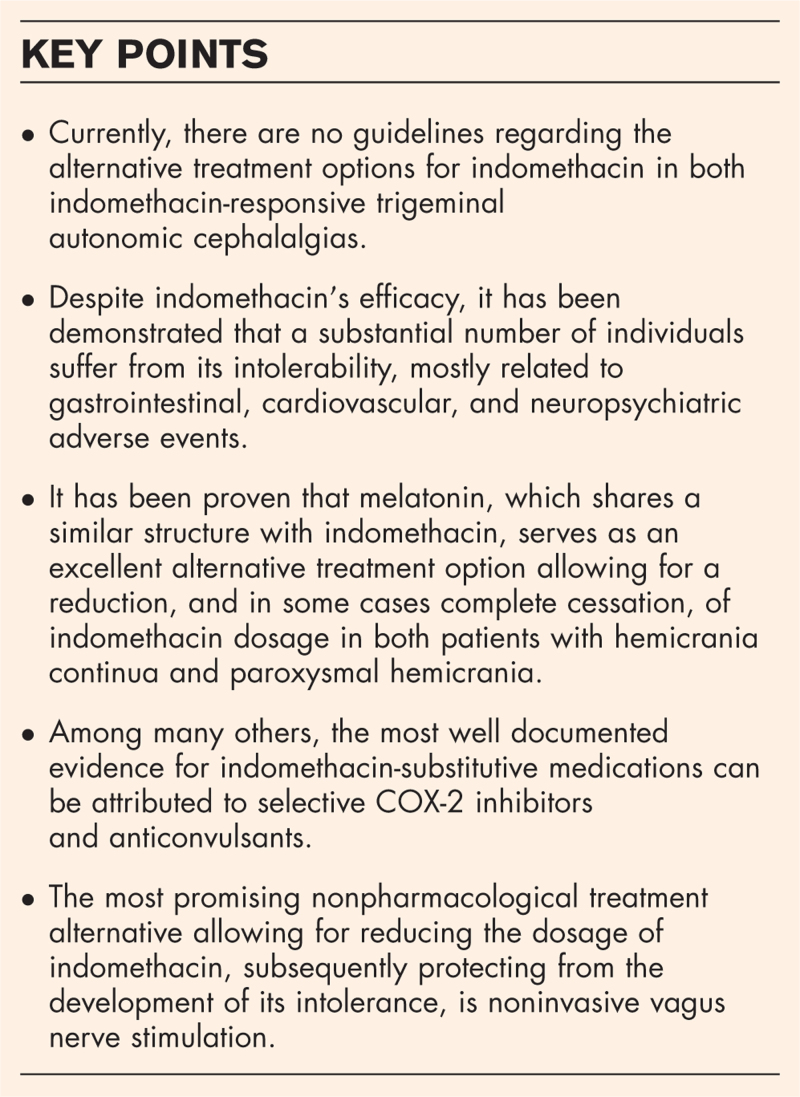
no caption available

Although indomethacin is considered a miracle drug for treatment of paroxysmal hemicrania and hemicrania continua, some patients may face challenges following the long-term indomethacin therapy [6,7]. Among many others, it has been proven that indomethacin is associated with higher risk of gastrolesive effects, specifically when compared to more commonly used NSAIDs [7,8]. As a result, a substantial number of individuals are forced to stop taking the medication due to the occurrence of indomethacin-related intolerance [6,7].

In 1992, A. Kuritzky [9] reported a first case of so-called ‘indomethacin-resistant hemicrania continua’, and since then, several other similar paroxysmal hemicrania and hemicrania continua cases have been described. However, the problematic of indomethacin-resistant cases is a subject of a more complex debate, as it is unclear whether a lack of response to indomethacin in headaches that are phenotypically compatible with paroxysmal hemicrania or hemicrania continua is a result of therapeutic insufficiency, or in fact is just a diagnostic failure [10].

Different pharmacological strategies for substituting indomethacin have primarily been documented only in case reports and case series, which has resulted in a dearth of sufficient guidelines and appropriate assessment of available therapy alternatives [7,10–12]. In light of the aforementioned events, this study aimed to review the relevant literature regarding the additional treatment options for indomethacin-responsive TACs in patients who demonstrated a complete abolishment of headache with indomethacin (in accordance with the current ICHD-III) [1]. Moreover, based on collected evidence, this study aimed to propose alternative treatment guidelines for patients who suffer from indomethacin intolerance.

## INDOMETHACIN: MECHANISMS OF ACTION AND SIDE EFFECTS

Apart from the well recognized cyclooxygenase inhibition, there are several other activities of indomethacin related to hemicranias’ pathomechanism, which may therefore be responsible for its tremendous efficacy in the treatment of paroxysmal hemicrania and hemicrania continua [7,13]. Those include reduction of cerebral blood flow (CBF), inhibition of nitric oxide pathways, and diminution of oxidative stress [7,13,14]. Additionally, indomethacin is believed to inhibit the relaxant effect of vasoactive intestinal polypeptide (VIP), substance P, or calcitonin gene-related protein (CGRP), which was demonstrated in the animal-based study by Vincent in 1992 [15]. As the blood levels of VIP and CGRP during exacerbation periods of hemicrania appear elevated [16], this targeted activity of indomethacin may play a crucial role in alleviating symptoms of indomethacin-responsive headaches.

Despite indomthecin's effectiveness, its activity is fraught with higher risk of gastrolesive effects when compared to other NSAIDs [8]. Apart from typical for NSAIDs gastrointestinal adverse reactions (nausea, dyspepsia, heartburn, ulceration, and bleeding) [17], prolonged indomethacin therapy carries increased cardiovascular risk [10], renal toxicity concerns [18^▪▪^], and alarming symptoms from central nervous system CNS [19,20^▪▪^]. The last commonly consists of fatigue (1–3%), dizziness (3–9%), and mood changes (1–3%) [20^▪▪^]. Rarely, the more concerning CNS side effects such as neuropsychiatric disturbance, hallucinations, paranoid psychosis, and even indomethacin-induced headache (especially in patients who have a personal or family history of migraine) may occur [6,19,20^▪▪^]. The consequence of all of the aforementioned is that when receiving indomethacin at conventional therapeutic dosages, 30–60% of patients report side effects, and 10–20% discontinue taking it entirely [7,21,22].

## MELATONIN

**FIGURE 1:**
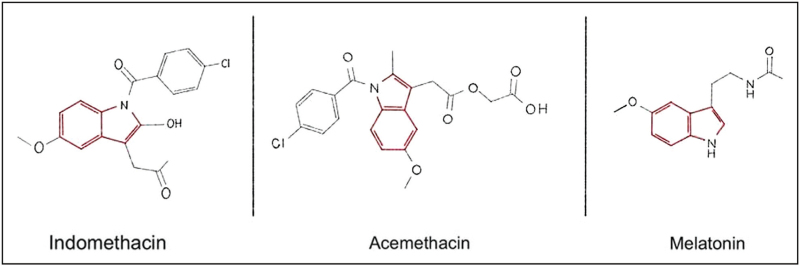
Left diagram - indomethacin, middle diagram - acemethacin, right diagram - melatonin.

Due to its feasible biological mechanisms of action and good safety profile, melatonin is among frequently used treatment choices. Melatonin is a pineal hormone sharing a similar structure to indomethacin, due to the presence of methoxy indole nucleus [23] (Fig. 1). There are several theories explaining the mechanisms of melatonin activity in headache disorders. Firstly, previous research has demonstrated the presence of melatonin receptors (MT1 and MT2) in the suprachiasmatic nucleus of the hypothalamus, which is involved in the pathophysiology of indomethacin-responsive TACs [7,18^▪▪^,^24^]. Secondly, melatonin's anti-inflammatory and antinociceptive properties have been shown to act as a free-radical scavenger, reducing the intensity of pain related to inflammation [18^▪▪^,^25^]. As a matter of importance, the supplementary use of melatonin could mitigate the gastrointestinal side effects of indomethacin, as it has been shown to provide protective mechanisms against peptic ulceration [26].

The role of melatonin in preventive treatment of indomethacin-responsive TACs has been well documented, especially regarding hemicrania continua individuals. Interestingly, a recent study also demonstrated its utility in paroxysmal hemicrania [18^▪▪^]. As demonstrated by Cheung *et al.*[18^▪▪^], in a sample including 23 hemicrania continua and 6 paroxysmal hemicrania individuals, melatonin proved its efficacy in a substantial cohort of patients, with a well tolerated safety profile. Hollingworth and Young [19] described a case of a woman whose hemicrania continua was associated with the occurrence of indomethacin-induced contralateral headache. Subsequently, indomethacin has been discontinued and replaced with melatonin 9 mg/night and brought a near-complete resolution of symptoms [19]. Spears [27] reported a patient who achieved a successful elimination of hemicrania continua attacks with 7 mg/night of melatonin, as indomethacin could no longer be tolerated due to gastrointestinal complications. Rozen [28] described a case of hemicrania continua individual, with indomethacin-related gastrointestinal side-effects, who remained pain-free on 9 mg/night of melatonin, and other individual who with 15 mg/night of melatonin was able to decrease indomethacin dosage from 150 mg to 75 mg/day. In another study including 11 hemicrania continua individuals [29], two patients managed to become pain-free and three were able to lower their dose of indomethacin by 50–75% with the help of 3–30 mg of melatonin.

## ACEMETHACIN

Nicpoń *et al.*[30] presented cases of three patients with hemicrania continua who were effectively treated with acemethacin. All individuals previously achieved an absolute response to indomethacin [30]. Subsequently, patients were treated with acemethacin in a retard form in a dose of 90 mg/day, with absolute efficacy [30]. Kikui *et al.*[31] reported a first Japanese case of hemicrania continua successfully treated with 180 mg/day of acemethacin.

Acemethacin, as a carboxymethyl ester of indomethacin (Fig. 1), holds equipotent anti-inflammatory inhibition effect on leucocyte inducible COX-2 enzyme and is characterized by a rapid bioconversion to indomethacin, which is its active metabolite [32]. Several studies demonstrated similar efficacy of indomethacin and acemethacin, however, in addition due to its lesser inhibitory effect on COX-1 enzyme, acemethacin appears to be better tolerated in the context of gastrointestinal side effects [33]. Interestingly, none of the cases reported by Nicpoń *et al.*[30] experienced any gastrointestinal adverse events in relation to acemethacin.

## OTHER NSAIDS

Therapeutic success of other NSAIDs is very limited and scarcely documented, as most of them are associated with barely none or minimal efficacy. Some case reports referred patients who achieved favorable outcomes with aspirin, naproxen, paracetamol, ibuprofen, and diflunisal [34–40]. The usefulness of piroxicam seems to be best observed, as it showed total responsiveness in few, both paroxysmal hemicrania and hemicrania continua patients, in doses of 20–40 mg/day [41,42].

## COX-2 SELECTIVE INHIBITORS

Although the exact mechanisms of action of COX-2 selective inhibitors in treatment of indomethacin-responsive TACs remain unknown, they may represent a good alternative to indomethacin. Peres *et al.*[43] presented a series of 14 patients with hemicrania continua treated with COX-2 inhibitors due to indomethacin-intolerance. As a result, 60% of patients who received celecoxib (200–400 mg/b.i.d.) and 33% receiving rofecoxib (50 mg/day) experienced a total response. Porta-Etessam *et al.*[44] presented a series of four hemicrania continua and one paroxysmal hemicrania patients who achieved total relief of pain with celecoxib, in doses ranging from 200 to 400 mg/day. Farag and Bahra [45] recently demonstrated the remarkable efficacy of etoricoxib in five patients (two paroxysmal hemicrania and three hemicrania continua), at total daily doses between 30 and 120 mg (median 90 mg) and celecoxib in one paroxysmal hemicrania patient with the dose of 400 mg/day.

In contrast to indomethacin, the COX-2 selective inhibitors preserve gastroduodenal COX-1 activity, and are therefore less likely to result in gastrointestinal side effects [45]. On the other hand, COX-2 inhibitors block the production of PGI2, which plays a crucial role as a platelet aggregation inhibitor and vasodilator, thus increasing a risk of a cardiovascular event [45,46]. Interestingly, Singh *et al.*[47] showed that during treatment with COX-2 inhibitors and indomethacin, a concomitant use of aspirin reduces a risk of acute myocardial infarction. In 2004, rofecoxib was world widely withdrawn on the basis of increased risk of cardiovascular events [48]. Nonetheless, Farag and Bahra [45] suggested that celecoxib and etoricoxib may be a safer alternative to indomethacin in terms of potential side effects, as indomethacin may have stronger adverse effects on blood pressure, platelet inhibition and cardiovascular dysfunction [49]. Interestingly, celecoxib seems to be associated with some of the lowest risk ratio for cardiovascular events, even when compared to naproxen, ibuprofen, and diclofenac [50]. According to a recent review [51], migraine patients treated with low doses of celecoxib present good treatment outcomes with relatively few undesirable events. Additionally, celecoxib as well as aspirin possess heart-protective features against myocardial hypertrophy and inflammation [52].

## ANTICONVULSANTS

Numerous research proved the efficacy of anticonvulsant drugs (especially gabapentin, pregabalin, and topiramate), making them a potential choice for sparing the dosage of indomethacin, especially in individuals facing indomethacin intolerance. The usefulness of gabapentin has been well described in hemicrania continua [53], as Spears [54] demonstrated in a series of hemicrania continua individuals, in which a significant majority noted at least 50% reduction of pain, and moreover, a substantial number of them became completely pain free. The dosage of the drug varied between 900 and 3600 mg/day [54]. Similarly, Kikui *et al.*[55] reported a series of four hemicrania continua patients who were able to substantially decrease their daily indomethacin dosage with 150 mg/day of pregabalin. Topiramate may be very beneficial for both paroxysmal hemicrania and hemicrania continua individuals suffering from indomethacin intolerance. Many authors reported highly successful treatment outcomes with topiramate at doses ranging from 50 to 200 mg/day, either as a support to indomethacin or as a solo therapy option [37,55–60]. Higher doses of topiramate should be avoided due to usually poor toleration [6,61].

## CALCIUM CHANNEL BLOCKERS AND ANTIDEPRESSANTS

The usefulness of calcium channel blockers (CCB) and antidepressants in both paroxysmal hemicrania and hemicrania continua appears controversial, as most of the cases presented in the literature represent individuals without a robust responsiveness to indomethacin. Furthermore, in the majority of instances, the efficacy of CCB and antidepressants is partial or even barely existent. According to Goadsby's clinical experience, verapamil seems useless in paroxysmal hemicrania [6].

## PROPOSED MANAGEMENT

If clinical presentation of a headache suggests the diagnosis of paroxysmal hemicrania or hemicrania continua [1], a careful physical examination should be performed. Physicians should be especially aware of potential secondary causes, that is why we recommend an MRI with pituitary view in all new encounters of these headache disorders [6,62^▪^]. In order to properly exclude pituitary dysfunctions, one should additionally perform testing for prolactin, insulin-like growth factor 1, and thyroid hormones [6,63,64]. Following the negative results, an indomethacin trial should be implemented. Indomethacin can be taken orally thrice daily (t.i.d.) for 5–7 days at a dose of 25 mg, then t.i.d. for 5–7 days at a dose of 50 mg, and finally t.i.d. for 2 weeks at a dose of 75 mg [6]. If there is a complete response at any moment, there is no need to escalate the dosage [6]. The diagnosis can also be established using a single-blind, placebo-controlled indomethacin test that is administered intramuscularly as 50, 100, or 200 mg (depending on patient's weight) of indomethacin, and placebo on alternate days [6,65]. Attack counts should be carefully recorded, and a sufficient untreated baseline period of five to seven days should be used to confirm the diagnosis [6]. Each administration of indomethacin should be supported with a gastrointestinal protection (e.g. proton pump inhibitor) [6,7]. Once the diagnosis is established, the indomethacin dosage should be lowered as much as possible, and the patient should be advised to determine their minimum efficacious dosage.

Remarkably, apart from pharmacological options, neuromodulation with noninvasive vagus nerve stimulation (nVNS) may appear beneficial for both indomethacin-responsive headache disorders allowing the reduction of indomethacin dosage [66^▪▪^]. The treatment with nVNS is usually very effective and well tolerated; however, it should be trialed for at least 3 months to assess the rate of effectiveness and to consider the potential necessity of adjusting the technique of stimulation [66^▪▪^].

**FIGURE 2:**
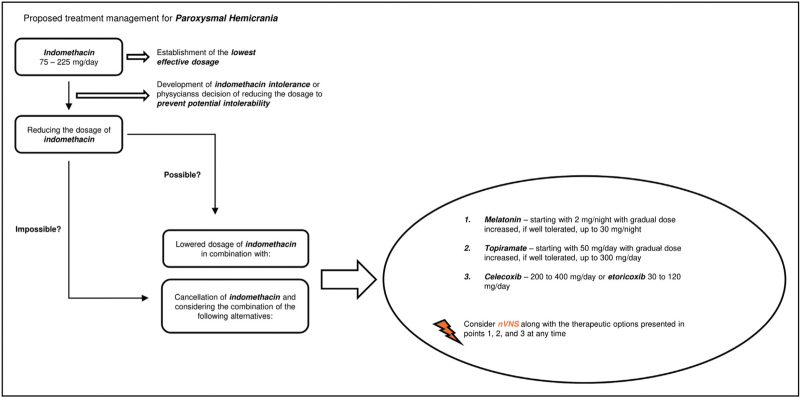
Proposed management for the treatment of paroxysmal hemicrania. nVNS, noninvasive vagus nerve stimulation.

**FIGURE 3:**
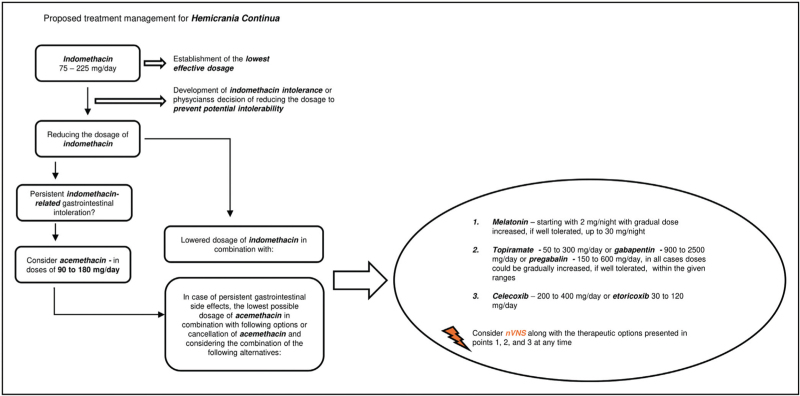
Proposed management for the treatment of hemicrania continua. nVNS, noninvasive vagus nerve stimulation.

In the clinical scenario of a patient developing indomethacin intolerance, or a physician's decision to reduce the administrative dosage of indomethacin to prevent probable intolerability and serious adverse reactions, one should consider alternative medication options. Based on collected clinical evidence, we propose the recommendations for substitutive treatment approaches. The suggested management for paroxysmal hemicrania can be seen on Fig. 2, while the suggested management for hemicrania continua is depicted on Fig. 3.

## FUTURE DIRECTIONS

Interestingly, González-Quintanilla *et al.*[67,68] recently described a case of a 68-year-old woman with hemicrania continua, who experienced a dramatic improvement of the headache following the administration of galcanezumab (initially 240 mg, then 120 mg/monthly). Nevertheless, great caution needs to be applied when interpreting the data, as authors indicate that although the patient noticed headache improvement with low doses of indomethacin, she did not show an absolute response to this medication (as it was poorly tolerated due to gastric discomfort), which undermines the correct diagnosis in respect to the ICHD, 3rd edition [67].

Regardless, considering the crucial role of the trigemino-vascular system activation with the release of pain-producing peptides (e.g. CGRP) in the pathophysiology in TACs, this class of drugs carries a possible potential worth investigating. It has been already demonstrated that CGRP antibodies are effective in treating episodic cluster headaches [69], and thus, there is a need for more clinical trials testing them in indomethacin-responsive TACs.

Promising results were recently achieved with Boswellia serrata extract (SBSE), which was tested on 37 hemicrania continua individuals, who demonstrated complete response to indomethacin [70]. Subsequently, SBSE was found to be not only exceptionally successful, but also incredibly well tolerated and free of serious side effects [70]. The use of the SBSE in the treatment of indomethacin-responsive headache disorders is still investigational, as more studies designed to assess its potential, efficacy, and safety are yet to be developed.

## CONCLUSION

Due to intolerance of its negative effects, many patients with hemicrania continua or paroxysmal hemicrania discontinue taking indomethacin. Acemethacin 90–180 mg/day may be a good alternative in hemicrania continua patients with preexisting gastric intolerance. Taking 2 mg of melatonin before bed and progressively increasing the dose as tolerated can be beneficial as a supplementary therapy in a substantial number of individuals. Celecoxib (200–400 mg/day) and etoricoxib (30–120 mg/day) may represent a good alternative to indomethacin; concomitant use of low-dose aspirin (75 mg/day) should decrease a risk of undesirable cardiovascular events. As for anticonvulsants, gabapentin 900–2500 mg/day and pregabalin 150–600 mg/day for hemicrania continua, and topiramate 50–300 mg/day for both paroxysmal hemicrania and hemicrania continua, make a good choice for indomethacin-sparing therapy, especially in those who suffer from its intolerability. For additional nonpharmacological support, nVNS may serve as a great alternative.

## Acknowledgements

*The data used in the manuscript have been provided by the original publications and are publicly available.*

### Financial support and sponsorship

*Dominik Taterra is supported by the Foundation for Polish Science (FNP) grant for young scientists.*

### Conflicts of interest

*None.*

## REFERENCES AND RECOMMENDED READING

Papers of particular interest, published within the annual period of review, have been highlighted as:

▪ of special interest
▪▪ of outstanding interest
